# User needs elicitation via analytic hierarchy process (AHP). A case study on a Computed Tomography (CT) scanner

**DOI:** 10.1186/1472-6947-13-2

**Published:** 2013-01-05

**Authors:** Leandro Pecchia, Jennifer L Martin, Angela Ragozzino, Carmela Vanzanella, Arturo Scognamiglio, Luciano Mirarchi, Stephen P Morgan

**Affiliations:** 1 Electrical Systems and optics research division, Faculty of Engineering, University of Nottingham, NG7 2RD, Nottingham, UK; 2 Hospital Trust S.Anna e S. Sebastiano, Caserta, Italy; 3 Italian Council of National Researches (CNR), Piazzale Aldo Moro 7, Rome, 185, Italy; 4 Hospital Trust Rummo, Benevento, Italy; 5 Siemens Healthcare Italy, Milan, Italy

**Keywords:** User needs elicitation, Analytic hierarchy process, AHP, Medical decision-making, Medical device

## Abstract

**Background:**

The rigorous elicitation of user needs is a crucial step for both medical device design and purchasing. However, user needs elicitation is often based on qualitative methods whose findings can be difficult to integrate into medical decision-making. This paper describes the application of AHP to elicit user needs for a new CT scanner for use in a public hospital.

**Methods:**

AHP was used to design a hierarchy of 12 needs for a new CT scanner, grouped into 4 homogenous categories, and to prepare a paper questionnaire to investigate the relative priorities of these. The questionnaire was completed by 5 senior clinicians working in a variety of clinical specialisations and departments in the same Italian public hospital.

**Results:**

Although safety and performance were considered the most important issues, user needs changed according to clinical scenario. For elective surgery, the five most important needs were: spatial resolution, processing software, radiation dose, patient monitoring, and contrast medium. For emergency, the top five most important needs were: patient monitoring, radiation dose, contrast medium control, speed run, spatial resolution.

**Conclusions:**

AHP effectively supported user need elicitation, helping to develop an analytic and intelligible framework of decision-making. User needs varied according to working scenario (elective versus emergency medicine) more than clinical specialization. This method should be considered by practitioners involved in decisions about new medical technology, whether that be during device design or before deciding whether to allocate budgets for new medical devices according to clinical functions or according to hospital department.

## Background

To provide high quality care for patients, the healthcare industry is dependent upon the provision of complex and expensive medical devices. It is widely accepted that if devices are to be used effectively they must meet the requirements of their users [1], however, capturing user requirements for healthcare technology is extremely complex. Although clinical effectiveness and safety are the primary concerns in medicine, many other aspects must also be considered including training needs, storage, labelling, servicing and cleaning [2]. Moreover, for the same medical device, the concepts of effectiveness and safety may change according to the specific clinical problem, medical specialization and patient condition.

The topic of user requirements of medical devices is of interest to a wide variety of individuals and organisations that are required to make decisions on the development, purchasing and prescription of these products. However, research has shown that collecting and considering this information is a challenging undertaking; a lack of time and resources may preclude rigorous work into requirements [3], as can a lack of knowledge of appropriate methods for data collection and analysis [4]. This can result in the collection of data that are incomplete, difficult to interpret or that fail to address the questions of interest [5].

Finally, and most fundamentally, the complex nature of medical device user requirements means that for any one medical device there are likely to be a large number of possible users, potentially including both professional and lay users, all with differing specialities, skills and abilities. Even within seemingly homogeneous user groups, individuals will have received different training and will vary in their working patterns, attitudes and preferences. In addition, how a device is used will vary considerably, according to the particular clinical procedure being performed and the physical and organisational context in which it is used [2]. This information must not only be collected and considered, but differences and conflicts between users must also be balanced. This is a critical issue for the developers of medical technology but also for healthcare providers when making purchasing decisions. It is a particular issue for publically funded healthcare providers who must demonstrate that the purchasing decisions about high-cost equipment are transparent and are be based on the best possible evidence available at the time.

The use of sc*ientific quantitative methods to support decision making* is considered necessary in healthcare organizations, where the personnel are committed to follow only the best available evidence according to well-designed trials [6], meta-analyses [7] or network meta-analyses [8]. Nonetheless, despite the hierarchy of evidence, the complexities of medical device decision-making require a spectrum of qualitative and quantitative information [9]. At the start of a user need elicitation problem, a wide-ranging and open-ended study should be conducted to collect data about the needs and priorities of healthcare professionals [10]. This type of information is critical to developing a broad understanding of the range of user requirements. In medical decision-making, qualitative methods have a crucial role in examining evidence from previous studies [9,11] and appraising this according to different contexts of use. It has been suggested that improving the methods used in qualitative studies will legitimise this type of data and increase its use in healthcare decision-making [12] as advocated by Kaplan [13], who concluded: “a *plea is made for incorporating qualitative/interpretive/subjectivist methods, without prejudice to other approaches”.* Furthermore, evidence-based care advocates that medical decisions are made with reference to the best available research evidence [14].

However, the nature of qualitative research can limit its use in scientific decision-making tasks such as user needs requirements elicitation for medical devices. The influence that the researcher plays in designing and interpreting studies has resulted in qualitative methods being viewed with scepticism by the medical community [15]. In addition, researchers have encountered problems when attempting to use qualitative data in the analytic and scientific decision-making processes that are a fundamental part of healthcare research [16]. For example, how can open-ended interview data collected from a number of caregivers with a range of opinions be used to make decisions on the design of a new medical device in a transparent and rigorous way [5]. There is need therefore for new approaches that allow the breadth and depth of the topics under investigation to be captured, yet also allow these to be quantified and prioritised, and for the process to be as transparent as possible. This is not only important for the decision makers but also for the healthcare staff; research has shown that successful adoption of new healthcare technology is dependent upon joint ownership of the decisions made during the development process [17]. Moreover, the decision outcome should be easy to understand, as intelligibility is strongly appreciated in medical domain decision-making [18-20], especially in the public sector. Finally, although not the primary aim of this study, the use of AHP clearly has implications for device manufacturers and future technology strategy in this area. In fact, medical device companies have also demonstrated an interest in scientific methods to elicit user needs, to enable them to respond to clinical demand and to enter new markets by adapting their products to the requirements of different medical specializations [21].

The Analytic Hierarchy Process (AHP) is a multi-dimensional, multi-level and multifactorial decision-making method based on the idea that it is possible to prioritize elements by: grouping them into meaningful categories and sub-categories; performing pairwise comparisons; defining a coherent framework of quantitative and qualitative knowledge; measuring intangible domains. This hierarchical approach allows the construction of a consistent framework for step-by-step decision-making, breaking a complex problem into many small less-complex ones that decision-makers can more easily deal with. This paradigm, known as *divide et impera*[22] (divide and rule) and widely investigated in medicine [23,24], has been demonstrated to be effective in healthcare decision-making [25].

The AHP is effective for quantifying qualitative knowledge as it allows intangible dimensions such as subjective preferences and comfort to be measured. This is important in medical decision-making as these factors [26], which are normally examined with qualitative research, cannot be measured directly using an absolute scale [27]. The AHP is particularly effective for quantifying experts’ opinions [28] that are based on personal experience and knowledge to design a consistent decision framework. This is a crucial point in any medical context [13], where not all of the relevant information is objective or quantitative. A number of researchers have highlighted the benefits of using AHP to explore user needs in healthcare [29,30], and in particular for including patient opinions in health technology assessment [31,32], choosing treatments [33], and improving patient centred healthcare [34,35]. Other methods that have attempted to elicit and quantify user needs in healthcare are conjoint analysis (CA) [36] , discrete choice experiments [37] and best-worst scaling [38]. A growing number of articles have focused on comparing AHP with these methods, and in particular with CA. According to Scholl et al. [39], AHP has proven to be more suitable than CA for complex decisions involving many factors. Mulye [40] suggested that AHP is more effective than CA when more than 6 attributes have to be prioritized. Ijzerman et al. [41] concluded that AHP, when compared with CA, resulted in more flexible, easier to implement and shorter questionnaires, although it may generate some inconsistences and other methods may have a more holistic approach. In another study, Ijzerman et al. [42], concluded that AHP lead to the overestimation of some alternatives although the differences found between AHP and CA, were mainly ascribed to the labelling of the attributes and the elicitation of performance judgments.

In our elicitation of user needs, we used AHP rather than the methods mentioned above because this method has been applied to medical decision-making [43] at the hospital level for budget allocation [44] and medical device purchasing [45]. It has been shown to be useful for a range of healthcare related decisions and for individuals from a range of backgrounds. As such, this method has the potential to be effective for the different organisations and individuals that are interested in eliciting user requirements, for example: developers wishing to improve device design, hospital managers who must allocate budgets and clinical engineers that are required to select devices. In addition to assisting each of these isolated tasks, a method that could be shown to be usable by all these groups could also improve communication between them, which is also essential in healthcare decision-making. AHP is normally used within a group decision-making process and requires that the decision-makers meet to compare and discuss their weights and decisions as a means to develop a consensus on group weights and achieve a group decision. However, this was not the purpose of this study, which aimed instead to explore the differences between the needs of clinicians with different specializations and different clinical settings. In summary, the adoption of a common method to elicit and prioritise user requirements could facilitate a wide range of decisions related to the design, selection and purchasing of medical devices.

In this study, we focus on clinical user needs related to the use of a multi-slice Computer Tomography (CT) scanner in a medium size city hospital. The multi-slice CT scanner refers to a special CT system equipped with a multiple-row detector array to collect simultaneously data at different slice locations. The multi-slice CT scanner has the capability of rapidly scanning a large longitudinal volume with high resolution. There are two modes for a CT scan: step-and-shoot CT or helical (or spiral) CT [46]. In recent years, developments in CT technology have provided increasing temporal and better spatial resolution. Scan times are much shorter and slice thickness much thinner with increasing rotation speed and increasing number of active detector-rows, from 4 and 16 detector rows to 64-detector CT scanners [47]. The different features of this device may significantly affect its costs. For instance, to equip this device with a system for continuous patient monitoring during the examination may be expensive. In addition, the technical performance of the device may strongly vary, affecting the final cost. It is therefore of paramount importance to elicit user needs before the purchasing decision is made to ensure that the right device is chosen and not one with unnecessary and costly features.

In particular, we focus on the application of AHP to identify the differences between the needs of clinical users, stratifying them according to specialization and intervention (elective versus emergency). We describe how the AHP method was adapted to improve its effectiveness for application in healthcare contexts [21,48], while a more general description of the AHP can be found elsewhere [49].

## Methods

### Ethical considerations

Before beginning the study the protocol was discussed with the hospital ethical committee. As this was an interview study with clinical staff and without patient involvement, no formal approval by an ethics committee was required. A participant information sheet was presented and discussed with participants before their involvement.

### Hierarchy definition

A focus group identified a total of 12 different clinical needs that must be satisfied by a CT-scanner. This focus group involved 4 medical doctors in charge of the units, of which 2 are co-authors of this paper (AR and AS), 3 biomedical engineers with extensive experience of the design, assessment and management of medical devices, of which 2 are co-authors of this paper (LP and LM) and 1 clinical engineer of the hospital. This group identified 12 needs, based on their personal experience and the pertinent scientific literature, and organized them into meaningful categories. LP acted as the facilitator and, based on his experience of AHP, designed the hierarchy, which was then reviewed with the other participants to check that it was accurate and comprehensive.

**Figure 1:**
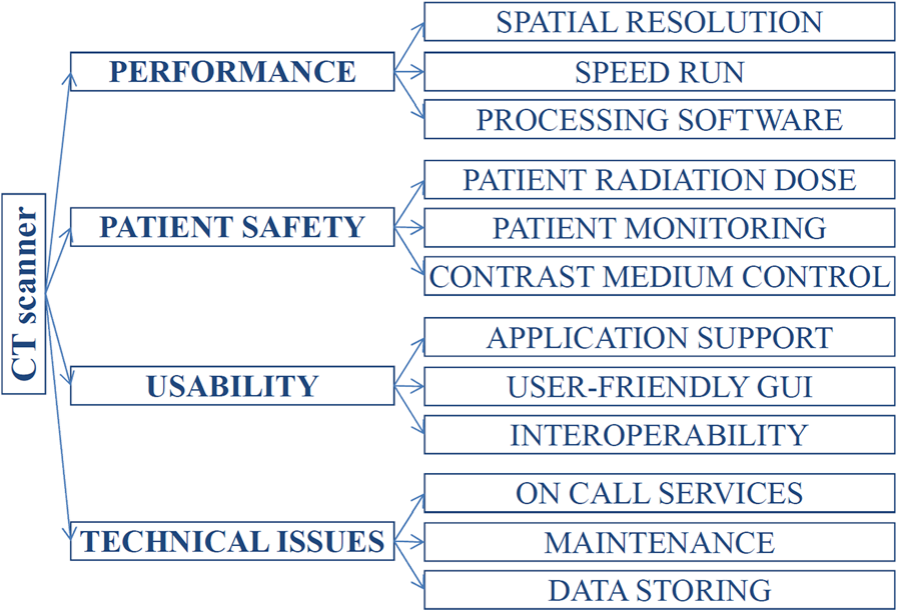
Tree of needs.

The 12 needs were organized into four categories and a tree was designed in which each node represented a category, and each leaf represented a need (Figure 1).

### Questionnaires

**Figure 2:**
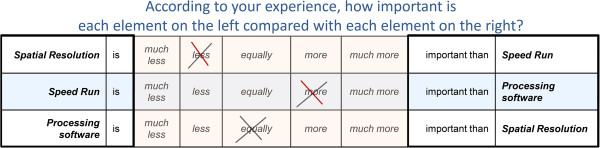
Questionnaire layout.

Questionnaires were designed to enable each respondent to compare the relative importance of each need with all of the other needs within the same category. The layout of the questionnaire is illustrated in Figure 2.

For each pair of needs (*ij*), responders were asked the following question: “in the selection of a new CT scanner, according to your experience, how important do you consider the element *i* compared to the element *j*?”. Responders answered by choosing one of the following judgments: much less, less, equally, more, or much more important. In accordance with the Saaty natural scale [50], an integer numerical value was given to each judgment: 1 if equally, 3 if more important, and 5 if much more important. The reciprocal values were given to the remaining judgments: 1/3 if less important, 1/5 if much less important. In-between numbers were used for in-between judgments. Although several scales have been proposed for this process [51-53], in this study an adaptation of the Saaty natural scale was used as it is easier to understand for responders who are not skilled in complex mathematics or with the AHP method. In this study, we used a three-point scale and not a nine-point scale as previous studies [27][54], involving approximately 200 responders unskilled in the use of AHP have shown that:

1. Although having a 9-point scale, most responders did not use more than 3 judgments (equal, more, much more) when comparing up to 4 elements.

2. Lay users reported confused when using a more complex scale.

Other studies have utilized a reduced scale (see supplementary material of [42]), although not clearly stated in the method section of the paper. After normalizing the eigenvectors by using the distributive mode [49], the results achieved with a five-point scale are equivalent to those achieved using the nine-point fundamental scale. These results were presented in four articles at recent International Symposia on AHP (ISAHP) [55,56] and [26].

The process was then repeated, designing similar questionnaires to elicit the relative importance of each category of needs. The questionnaire was designed to minimize possible responder bias. As responders writing from left to right and top-down can be more likely to judge the elements on the top-left as more important than those on the bottom right, each element was presented the same number of times on the left and the right, at the top and at the bottom of the questionnaire Moreover, the sequence of comparisons (A with B, B with C and C with A) was adapted to minimize intransitive judgments [54].

### Judgment matrix

For each category of needs, a judgment matrix A_*nxn*_ was designed, where “n” is the number of needs in this category. According to Saaty theory [50], each matrix had the following properties:

1. The generic element (a_ij_) referred to the ratio between the relative importance of the need “*i”* (N_i_) and “*j”* (N_j_);

2. The element a_ji_ was the reciprocal of a_ij_, assuming the reciprocity of judgment (if N_i_ was 3 times more important than N_j_, then N_j_ should be 1/3 of N_i_);

3. The element a_ii_ was equal to 1 (N_i_ is equal in importance to itself);

4. The matrix A was assumed to be a transitive matrix, which means that “∀ *i*, *j*, *k* **∈** (1; *n*), *a*_*ij*_ = *a*_*ik*_ * *a*_*kj*_” by definition of a_ij_ (see Equation 1).

(1) $$a_{\mathrm{ij}}=\frac{N_{i}}{N_{j}}=\frac{N_{i}}{N_{k}}*\frac{N_{k}}{N_{j}}=a_{\mathrm{ik}}*a_{\mathrm{kj}}$$

This last property is called the transitivity property and reflects the idea that if “i” was considered twice as important as j (N_i_= a_ij_ * N_j_), and “j” was considered three times more important than “k” (N_j_= a_jk_ * N_k_), then “i” should be judged six times (two times three) more important than “k” (N_i_ = a_ik_ * N_k_, with a_ik_=a_ij_* a_jk_).

### Local weights: the relative importance of needs within each category

It has been proved [50] that, if a matrix A satisfies the properties described in section 2.4 then each column is proportional to the others and only one real eigenvalue (λ) exists, which is equal to “n”. The eigenvector associated with this eigenvalue is again proportional to each column, and represents the relative importance of each need compared to each of the other needs in the same category. The relative importance (weight) of a need *i* within the category *m* will be further recalled as LW_i_^m^ or local weight.

In cases where the judgments are not fully consistent, the columns of the matrix are not proportional to one another. In addition, the matrix has more eigenvectors and none are proportional to all the columns. In this case, the main eigenvector, which is the one corresponding to the largest eigenvalue (λ_max_), is chosen. Its normalized components represent the relative importance of each need.

### Consistency estimation

If the transitivity property is not respected, an inconsistency will be generated. This inconsistency was estimated by posing some redundant questions. Considering three needs (*i*, *j*, and *k)* the respondent was asked to perform the pair comparisons *ij* and *jk*, and then the redundant comparison *ik*. The answer to the redundant question was compared with the one deduced from the first two, assuming the transitivity of judgment. The difference between the real answer and the transitive one represents the degree of inconsistency. The global effect of this inconsistency was estimated by measuring the difference between the major eigenvalue λ_max_ and “n”. The error is zero when the framework is completely consistent. Inconsistency is, in the majority of cases, due to loss of interest or distraction. If inconsistency occurs, the responders are required to answer the questionnaire again. Some inconsistency between responses is expected; using a scale of natural numbers will cause some systemic inconsistency because not all the ratios can be represented and because of the limited upper value (e.g. 3*2 gives 6, but the maximum value in the scale is 5). For this reason, an error less than a certain threshold was accepted in accordance with the literature [57]. An error over this threshold should be considered too high for reliable decisions.

At each node, the responders’ consistence was estimated measuring the difference of the eigenvalue λ_max_ from “n” (number of elements in the node), normalized to “n”. This is defined as the consistency index (CI) [57], and is zero when the framework is completely consistent (λ_max_=n). According to literature, the CI is divided by the Random Consistency Index (R.I.), which is a tabled [57] value changing for *n* from 1 to 9. This ratio is called Consistency Ratio (CR=CI/CR) and a threshold of CR≤ 0.1 is generally considered appropriate, although some authors have proved that it is possible to increase this threshold to 0.2 when the hierarchy is complex and it is not practical for the responders to discuss the questionnaire results [26,54].

### Category importance per responder

By applying the same algorithm to the categories it was possible to evaluate their relative importance. The relative importance of a category *m* will be further recalled as category importance (weight) or Categorical Weight (CW^m^).

### Global-importance of each need per responder

Finally, the relative importance of a need *i* compared to all the others (not only those in the same category) is defined as global-importance (Global-Weight) of the need *i* (GW_i_). GWs are calculated by multiplying the local (within category) importance of the need by the importance of the root element (category) into the Hierarchy. For instance the global-weight of the need *i*, which is in the category *m*, was calculated as the product of the local importance of the need (LW_i_^k^) and the importance of its category *m* (CW^m^) (Equation 2).

(2) $$GW_{i}=LW_{i}^{k}*CW^{k}$$

### Correlations among responders’ preferences

The goal of this study was to explore the differences between user needs for a CT scanner, stratifying clinicians according to specialization and type of intervention (elective versus emergency), and not to find consensus between them. Finding consensus usually requires that the group of responders meet to compare and discuss their weights and to agree a group decision. Nonetheless, this study did investigate the correlations between the responses to understand whether needs were more homogeneous according to clinical specialization (i.e. neurologists versus ear surgeon) or according to the type of intervention (elective versus emergency). This is an important issue both for device design and purchasing.

Several methods have been proposed to measure consensus [58], but as stated, this study does not aim to obtain a consensus, but rather to measure correlations to investigate differences in the needs of different users. Thus, the Spearman rank correlation (ρ or RHO) was calculated, as this measure is widely used for AHP-based studies [54,59]. This correlation measures mathematically if two sets of elements are ranked in the same order [39]. Large values of RHO show well-matched rankings (1, identical ranking) of prioritized elements. To verify the significance of ρ, the p-value was used to test the hypothesis that two responders’ prioritizations are meaningfully correlated. A value of p less than 0.05 was considered significant, according to existing literature [54]. Thus, the homogeneity of correlations was tested by calculating the matrix of p-values for testing the hypothesis of no correlation against the alternative that there is a nonzero correlation. Each element of this matrix is the p-value for the corresponding element of RHO. If the p-value (i, j) is less than 0.05, then the correlation RHO (i, j) is significantly different from zero, which in this study meant that responder-i and responder-j prioritized the need in the same order.

### User feedback

Finally, to fully understand the reasons behind the needs prioritization, the results obtained were discussed with the responders, other domain experts (clinicians working in similar scenarios to the responders) and the Medical Director of the Trust. Some open questions were also posed to obtain feedback on the method.

### Responders

Five clinicians (age 54±5 years, 40% males), each with more than 20 years of experience, working in the same medium-sized public hospital, were the final responders in the study and completed the questionnaires. None of these clinicians was one of the authors of this paper. All had experience of different clinical environments, but each was asked to answer in relation to the unit in which they were working at the time of the study, which were: radiology unit, emergency unit, minimally invasive ear surgery unit, neurology unit. The surgeon from the ear surgery unit was mainly responsible for child ear cochlear implants, which is an elective surgery. Two surgeons answered from the neurology units: one was in charge of emergency neurological surgeries and the other of the elective neurologic surgeries.

## Results

The relative importance for each category of needs is reported in Table 1.

**Table 1:** Categorical local weights (CR≤0.1)

	**radiology**	**ear surgery**	**neurology**	**emergency neurology**	**emergency**
**PERFORMANCE**	0.22	**0.44**	**0.39**	0.19	0.18
**SAFETY**	**0.48**	0.34	0.29	**0.57**	**0.60**
**USABILITY**	0.19	0.14	0.22	0.17	0.13
**TECHNICAL ISSUES**	0.11	0.08	0.10	0.08	0.09

The global and local weights of each need are reported in Table 2.

**Table 2:** Local and global weight of needs (CR≤0.1)

	**radiology**		**ear surgery**		**neurology**		**emergency neurology**		**emergency**	
	**GW**	**(LW)**	**GW**	**(LW)**	**GW**	**(LW)**	**GW**	**(LW)**	**GW**	**(LW)**
**PERFORMANCE**										
Spatial Resolution	**.07**	(.32)	**.28**	**(.64)**	**.19**	(**.48)**	**.08**	**(.43)**	.05	(.30)
Speed Run	**.10**	**(.46)**	.05	(.11)	.04	(.11)	**.08**	**(.43)**	**.09**	(**.52**)
Processing software	.05	(.22)	**.11**	(.26)	**.16**	(.41)	.03	(.14)	.03	(.18)
**SAFETY**										
Patient radiation dose	**.16**	(.33)	**.11**	(.33)	**.10**	(.33)	**.08**	(.14)	**.11**	(.18)
Patient Monitoring	**.16**	(.33)	**.11**	(.33)	**.10**	(.33)	**.33**	**(.58)**	**.40**	**(.66)**
Contrast medium control	**.16**	(.33)	**.11**	(.33)	**.10**	(.33)	**.16**	(.28)	**.10**	(.16)
**USABILITY**										
Personnel Education	**.13**	(**.69**)	**.07**	**(.52)**	**.07**	(.32)	**.08**	(**.48**)	.04	(.33)
User-friendly GUI	.04	(.23)	.03	(.18)	.05	(.22)	.02	(.11)	.04	(.33)
Interoperability	.02	(.08)	.04	(.30)	**.10**	**(.46)**	**.07**	(.41)	.04	(.33)
**TECHNICAL ISSUES**										
Technical Assistance	.04	(.33)	.03	(.33)	.02	(.22)	.05	(**.66**)	.04	(**.46**)
Maintenance	.04	(.33)	.03	(.33)	.03	(.32)	.01	(.16)	.03	(.32)
Data Storing	.04	(.33)	.03	(.33)	.05	(**.46)**	.01	(.18)	.02	(.22)

Table 3 and Table 4 show the relationship between the responders’ prioritization via Spearman rank correlation, according to respectively per category weight and per needs’ global weight.

**Table 3:** **Spearman correlation** (*ρ*) **and p-value** (*p*) **among responders per categories prioritization**

	**Radiology**	**ear surgery**	**Neurology**	**Emergency neurology**	**Emergency**
	***ρ(p*)**	***ρ(p*)**	***ρ(p*)**	***ρ(p*)**	***ρ(p*)**
**radiology**	1	-	-	1(0.040)	1(0.040)
**ear surgery**		1	1(0.040)	-	-
**neurology**			1	-	-
**Emergency neurology**				1	1(0.040)
**emergency**					1

**Table 4:** **Spearman correlation (ρ) and p-value *(p) *among responders per needs’ GW**

	**Radiology**	**ear surgery**	**Neurology**	**Emergency neurology**	**Emergency**
	**ρ(*p*)**	**ρ(*p*)**	**ρ(*p*)**	**ρ(*p*)**	**ρ(*p*)**
**radiology**	1	0.73(0.004)	-	0.82(0.001)	0.77(0.002)
**ear surgery**		1	0.86(0.000)	0.74(0.003)	0.61(0.018)
**neurology**			1	-	-
**Emergency neurology**				1	0.90(0.000)
**emergency**					1

All responders achieved the required threshold for coherence (CR≤0.1), as detailed in Table 5.

**Table 5:** Consistency ratio (CR) per responder per questionnaire

	**radiology**	**ear surgery**	**neurology**	**emergency neurology**	**emergency**
**Questionnaire 1: PERFORMANCE**	0.00	0.04	0.03	0.00	0.01
**Questionnaire 2: SAFETY**	0.00	0.00	0.00	0.01	0.03
**Questionnaire 3: USABILITY**	0.10	0.01	0.00	0.03	0.00
**Questionnaire 4: TECHNICAL ISSUES**	0.00	0.00	0.00	0.03	0.00
**Questionnaire 5: CATEGORIES**	0.01	0.07	0.06	0.04	0.02

## Discussion

In this paper, we presented the results of a study on the application of AHP to elicit clinical user needs. As a case study, we focused on user needs related to the use of a CT scanner in a medium size hospital.

For elective surgery (ear and neurology), technical performance was considered the most important category of needs, while in emergency departments the safety of the patient was the dominant need. Patient safety was considered at least the second most important category by all the clinicians. All the responders considered technical issues the least important category. The results in Table 1 show that the relative importance of each category of needs varied according to the type of intervention rather than for the clinical specialization. This is illustrated by the strong and statistically significant correlation between the priorities of the neurologist performing elective surgery and the surgeon in charge of ear cochlear implants in children (Table 3). Discussion of the results with the responders confirmed that their needs were the same: first scanner performance (in both cases anatomical details and processing capability were crucial), then patient safety (an issue which is a priority for the whole medical field), usability and finally technical issues (considered important but not as much as the other needs). Table 3 demonstrated that no significant rank correlation was observed between the neurologists performing elective and emergency surgeries. Finally, the rankings between surgeons working in emergency departments were strongly and significantly correlated (Table 3). Discussion of these results with responders confirmed that their needs were the same: first patient safety (due to the unstable condition of the majority of their patients), then performance (execution time was crucial, once again due to patient instability), then usability and finally technical issues. The clinician in charge of the radiology unit ranked the need categories similarly to the emergency surgeons, but with different motivations: first patient safety (as a general medical approach, but also because of legal responsibility), then performance (to address working organization, unit competitiveness and radiologist scientific interest), usability and technical issues.

Regarding local weights within the category of *Performance* (Table 2), in elective surgeries, spatial resolution was considered the most important need. This reflected the fact that there are similarities between neuro-surgeries and cochlear implantations in terms of the need to investigate small anatomical details. For this type of case, the neurologist considered the processing software almost as important as the spatial resolution, reflecting the fact that the images used for neurology surgery require more complex pre- and post- processing than those for ear implants. Speed was not considered crucial mainly because the patients undergoing this procedure are usually stable. Again regarding the performance, in emergency surgeries, speed run was considered of paramount importance due to the unstable condition of the patients, which placed them at risk of death or serious impairment. The neurologist reported that spatial resolution was as important as speed run, due to the importance of anatomical details in neurosurgery. Processing software was reported as the least important issue as in emergency situations real-time information is crucial and software requires time to process images. The prioritization of the radiologist was more similar to the rankings of emergency surgeries than elective. Once again, by discussing this result with the Trust Medical Director, it emerged that the majority of radiologist activities are requested from the emergency unit and therefore the daily activities of the radiologist influenced his priorities.

Regarding the local weights in the category of *Safety* (Table 2), in elective surgeries as in radiology, all the issues were considered equally important. This is likely to be due to the fact that patient safety is an important issue in all branches of medicine. However, it takes on even greater importance in emergency situations, and this was reflected by the differences in importance between needs in the safety category for emergency surgery. Patient monitoring was scored as most important, as patients are frequently in unstable conditions during these kinds of surgeries. The neurologist also considered contrast medium control as important as the brain is particularly sensitive to these drugs. Radiation dose was considered less important during emergencies as the critical nature of these procedures justify some risk to the patient from radiation exposure.

The highest variation in local weights of needs was found in the category of *Usability* (Table 2). This reflects different needs with regard to this factor; the radiologist, the surgeon responsible for cochlear implants and the emergency neurologist scored application support as the most important need. The neurologists considered interoperability important, for both emergency and elective surgeries. This reflected the fact that they often needed to integrate information from images obtained with different technologies (ultrasound, magnetic resonance and CT).

Regarding local weights of needs in the *Technical issues category* (Table 2), no significant information emerged from the radiologist and the ear surgeon. The elective neurologist considered data storing important. In emergency, technical assistance was considered of paramount importance. Discussing this result with the emergency surgeons revealed that time to first intervention, up time and mean time to repair were considered important to guarantee service continuity. These were not considered crucial for elective surgery, where the number of interventions in the year and the condition of the patients meant that some delays were acceptable.

Regarding global weights, Table 2 shows that for elective surgery the top five important needs are the same: spatial resolution, processing software, radiation dose, patient monitoring, and contrast medium. Similarly, in emergency surgery, the top five needs were the same: patient monitoring, radiation dose, contrast medium control, speed run, spatial resolution. Table 4 shows that there was again a higher rank correlation according to surgery, election-election (86%) or emergency-emergency (90%), more than according to specialization: neurologist-neurologist (ρ<50% and p>0.05). In addition, radiologist prioritization was significantly and strongly correlated to emergency (82% and 77% with p<0.01) more than to elective surgery. In this case, a significant correlation between radiologist and ear surgeon (73%, p<0.1) was observed. This result was unexpected considering that the number of CT scans required for ear-surgery represents less than the 5% of the total activity of radiology. From a methodological point of view, this result was mainly due to the fact that both the radiologist and the clinician responsible for ear-surgery scored all the needs in safety and technical categories as equally important. This could be a weakness of this method. Nonetheless, after discussing this result with the radiologists and with the Medical Director of the Trust, it emerged that this strong correlation was likely to be due to the fact that radiologist and ear surgeons had collaborated in designing surgery for cochlear implants and in this kind of intervention, computer assisted design in pre-surgery planning is crucial to select the cochlear device and to plan the implant. This may illustrate the strength of the AHP method in mapping specific needs of specific trusts.

Regarding the method, it should be noted that AHP is normally used within a group decision-making process and requires that the decision-makers meet to compare and discuss their weights and decisions as a means to develop a consensus on group weights and achieve a group decision. However, this was not the purpose of this study, which aimed instead to explore the differences between the needs of clinicians with different specializations and different clinical settings. We have demonstrated that there was high consensus between those clinicians working in similar settings (emergency versus elective medicine), independent of their clinical specialization. Regarding the usability of the method, all of the responders reported that they encountered no difficulties in completing the questionnaires and that the results accurately reflected their needs. Moreover, all declared that they would not have been able to spontaneously quantify their preferences in such a detailed manner. Furthermore, all five responders declared that the method helped them to elicit their needs. The other domain experts involved in this study found the method clear and useful for facilitating the user needs elicitation process. Limiting the number of elements in each category to three assisted the responders, who were not experienced with this method, particularly in avoiding inconsistency and speeding up the process. The scale used, from 1 to 5 and not to 9 as proposed by Saaty [50], resulted in more significance to responders, as already stated in previous research [26]. This was possible because of the low number of elements in each node. The careful design of the questionnaires facilitated responders’ coherence, which has been identified as an important issue in avoiding inconsistencies by other AHP studies in healthcare [41,42], especially when responders are patients. This is because AHP requires that the words used are familiar to lay responders and therefore care must be taken when naming needs and categories. Although, in this study, the responders were clinicians with extensive experience of the topics and terms under investigation it is still important to reduce the risks of confusion or misunderstanding.

This study supports the results of previous studies [28] that using a limited number of elements in the same node of the hierarchy may reduce inconsistencies. This study confirms the results of previously published papers [54] that less than five elements per node can be considered a satisfactory threshold to achieve a good level of significance. In addition, a reduced number of possible judgments, a 1 to 5 scale instead of a 1 to 9 one, reduced inconsistencies [28].

Therefore, to apply AHP method in a healthcare context, especially when patient and lay users are involved, we recommend: (1) the use of a limited number of possible judgments, for example a 1 to 5 scale, and (2) to put no more than 4 elements in each node. This last recommendation may require a deeper hierarchy, but it has been demonstrated that by adding more levels, the total number of questions is globally reduced [57].

Regarding the limitations of this study, the number of responders was relatively small, which means that it was not possible to investigate whether preferences for CT scanning varied, for example according to factors such as age, length of clinical experience and educational background. In addition, it also means that it is not possible to generalize the results to different scenarios such as different hospitals. Regarding the method, although according to the pyramid of evidence, studies basing on opinions are not considered the most reliable, a gap exist between evidence and every-day decision making healthcare organizations. AHP may contribute to combine empirical evidence and subjective experience in order to improve medical decision-making.

## Conclusion

User needs elicitation is a fundamental part of device design and purchasing. The method described in this paper allowed user needs to be elicited according to different working scenarios and medical specializations. Moreover, AHP provided an understandable and traceable framework for the decision process, which is essential in the public sector where decision makers are required to justify their choices to different stakeholders. This paper has demonstrated that, for this case study of a CT scanner, user requirements varied more according to medical scenario (elective surgery versus emergency) than to clinical specialization. This should be considered before when deciding whether to allocate budgets for medical devices according to clinical functions or according to hospital units. These results also have important implications for the manufacturers of CT scanners as they suggest that decisions on device functionality and features should be made according to the medical scenario rather than the clinical specialization. This would then enable manufacturers to produce competitively priced devices, which are appropriate for the particular clinical setting. The study also has wider implications for the medical device industry as it describes a rigorous and effective method for eliciting user requirements during the development of new devices. Finally, when using AHP in healthcare, two issues should be considered: firstly, to use a limited number of items in each node, and secondly, to use a limited scale for responders’ judgment.

## Competing interests

The authors declare that they have no competing interests.

## Authors’ contribution

LP, AR and LM conceived this study. LP and CV drafted the hierarchy and the questionnaires, analysed the data and presented the results. LP, AR, AS, LM participated to the focus group, reviewed the hierarchy of factors, prepared the ethical application, enrolled the responders, coordinated the elicitation study, submitted the questionnaires, discussed the results with other medical personnel. LP, JLM and SPM discussed the results considering the state of the art of the literature, drafted the paper and reviewed the manuscript. All the authors contributed to the paper. All authors read and approved the final manuscript.

## Pre-publication history

The pre-publication history for this paper can be accessed here:

http://www.biomedcentral.com/1472-6947/13/2/prepub
